# Microbiota–Gut–Brain Axis as a Therapeutic Nexus: Multimodal Probiotic, Antidepressant, and Nutritional Strategies

**DOI:** 10.3390/microorganisms14102384

**Published:** 2026-10-18

**Authors:** Jiangang Lv, Jiayi Xu, Shuojie Lv, Qinwen Zheng, Yixin Chen, Qingjing Wang

**Affiliations:** 1 Key Laboratory of Artificial Organs and Computational Medicine of Zhejiang Province, Key Laboratory of Pollution Exposure and Health Intervention of Zhejiang Province, Shulan International Medical College, Zhejiang Shuren University, Hangzhou 310015, China; 2 Keyi College, Zhejiang Sci-Tech University, Shaoxing 312369, China; 3 College of Biology and Engineering, Zhejiang Shuren University, Hangzhou 310006, China; 19057144330@163.com (J.X.);

**Keywords:** major depressive disorder, probiotic, gut–brain axis, stress, diet, gut microbiota, nerve

## Abstract

Major depressive disorder (MDD) is a highly prevalent psychiatric illness worldwide, yet current therapeutic approaches still face challenges, including limited treatment efficacy, high interindividual variability, and high rates of treatment resistance. In recent years, the mechanisms by which the gut microbiota participates in emotional regulation and neuropsychiatric disorders through the “microbiota–gut–brain axis” have drawn increasing attention. Accumulating evidence indicates that influencing factors of depression, such as stress and diet, can alter gut microbial richness. Experimental studies have shown that gut microbes can modulate chemical signaling in the brain, induce neuroinflammation, and thereby affect the development and progression of depression. In humans, probiotic supplementation has shown promising antidepressant-like effects in preclinical models and some clinical trials, but its efficacy remains heterogeneous, appears to be strain-specific, and has not been consistently reproduced in placebo-controlled trials; it should therefore be considered an adjunctive or investigational approach requiring further validation before it can be recommended as a standalone or preventive intervention. This review will integrate findings from animal experiments demonstrating the preventive and therapeutic roles of probiotics and provide a critical overview of current research. To enable more effective translation of microbiota–gut–brain axis research into clinical trials and the use of probiotics as novel therapies, future studies should pay greater attention to the complex bidirectional communication between the gut and the brain. The brain influences gut microbes through neuroendocrine pathways, whereas gut microbes act back on the brain via vagal and immune modulation. Future research should also focus on the parallel effects of microbes on depression through neural, immunological, and metabolic routes. Clinical evidence remains heterogeneous, and probiotic or microbiota-targeted approaches should currently be interpreted as adjunctive or investigational rather than established standalone therapies.

## 1. Introduction

Major depressive disorder (MDD) is a common psychiatric illness characterized by persistent low mood, diminished interest, and impaired cognitive function, and it represents a major public health concern worldwide [1]. Major depressive disorder (MDD) has become a major public health problem, leading to disability and suicide. Although existing antidepressants are widely used in clinical practice, approximately one-third of patients still have a poor response to standard treatment, suggesting the limitations of traditional therapeutic strategies. In recent years, with the introduction of the concept of the “microbiota–gut–brain axis” (MGBA), research has gradually revealed the critical roles of the gut microbiota in emotional regulation, neuroinflammation, and stress responses. The gut microbiota not only participates in the synthesis and metabolism of neurotransmitters but also influences central nervous system functions through immunomodulation, endocrine signaling, and vagal pathways and is thus closely associated with the development and progression of depression [2]. More importantly, a growing body of research has confirmed that interventions targeting the gut microbiome—such as probiotic supplementation, dietary optimization, and pharmacological modulation—can significantly improve host emotional states and neurological function, making them important adjunctive therapeutic strategies for depression [3,4]. Accumulating evidence has revealed that interventions directed at the gut microbiota—including probiotic administration, dietary restructuring, and pharmacologic regulation—are capable of markedly ameliorating affective states and neurological function in the host, thereby positioning such approaches as valuable adjunctive modalities in the management of depressive disorders; among these, probiotics, particularly strains belonging to the genera *Lactobacillus* and *Bifidobacterium*, have been demonstrated to exert antidepressant-like actions via multifaceted mechanisms, including the reinforcement of intestinal epithelial barrier integrity, the attenuation of systemic inflammatory responses, and the modulation of central and peripheral neurotransmitter concentrations [5,6]. In parallel, prudent dietary regimens, exemplified by the Mediterranean diet and the ketogenic diet, attenuate symptoms associated with affective disorders by restructuring the gut microbiota composition [7]. In addition, select antidepressant pharmacotherapies have been demonstrated to inherently exert regulatory effects on the gut microbial ecosystem, with their clinical efficacy being partially attributable to such microbiome-directed modulation—an observation that underscores the potential relevance of drug–microbiome crosstalk in the therapeutic landscape of depression [8]. The present review aims to provide a systematic synthesis of the accumulated evidence pertaining to probiotic, pharmacological, and dietary approaches in the regulation of gut microbial ecology and the amelioration of depressive symptomatology, thereby providing a theoretical foundation and delineating future research avenues for microbiota-oriented interventions in the management of depression. This article is a narrative review. An updated targeted search of PubMed was conducted through 9 September 2026, using combinations of “major depressive disorder”, “microbiome and microbiota-gut-brain axis”, and “probiotic, prebiotic, synbiotic, antidepressant, diet, stress, vagus, inflammation and intestinal barrier”. The search was limited to English-language articles published between January 2000 and September 2026. As this is a narrative rather than a systematic review, no strict study-design filter was applied; original research, systematic reviews, and meta-analyses were all considered, whereas conference abstracts, editorials, and non-peer-reviewed preprints were excluded. Records were screened by title and abstract, followed by full-text assessment of potentially relevant articles. Priority was given to studies providing direct mechanistic evidence (e.g., germ-free models, fecal microbiota transplantation, vagotomy, or strain-specific supplementation), quantitative human associations between microbial taxa or metabolites and depression, or intervention trials with mood-related outcomes. Reference lists of retrieved reviews were also screened to identify additional key studies. Where evidence was conflicting or derived mainly from animal models, this is noted in the relevant sections.

## 2. The Gut Microbiota and the Pathogenesis of Depressive Disorders: Underlying Mechanisms

The pathogenetic involvement of the gut microbiota in depressive disorders is characterized by considerable complexity and multifacetedness, encompassing immune–inflammatory responses, intestinal epithelial integrity, endocrine homeostasis, and neural communication cascades. Rather than constituting a singular etiological determinant of depression, microbial dysbiosis exerts its effects in a synergistic manner across an array of interconnected biological circuits. To this end, the following discussion will provide a systematic exposition of the pathophysiological roles of the gut microbiota in depression, structured along five principal axes: neurotransmitter metabolism, neuroinflammatory processes, breakdown of the intestinal barrier, dysregulation of the hypothalamic–pituitary–adrenal (HPA) axis, and vagal afferent signaling. Among them, clinical trials and systematic reviews provide stronger evidence for intervention effects, human observational studies mainly support associations, and animal or in vitro studies primarily generate mechanistic hypotheses.

### 2.1. Modulation of Neurotransmitter Biosynthesis and Metabolic Turnover

**Figure 1:**
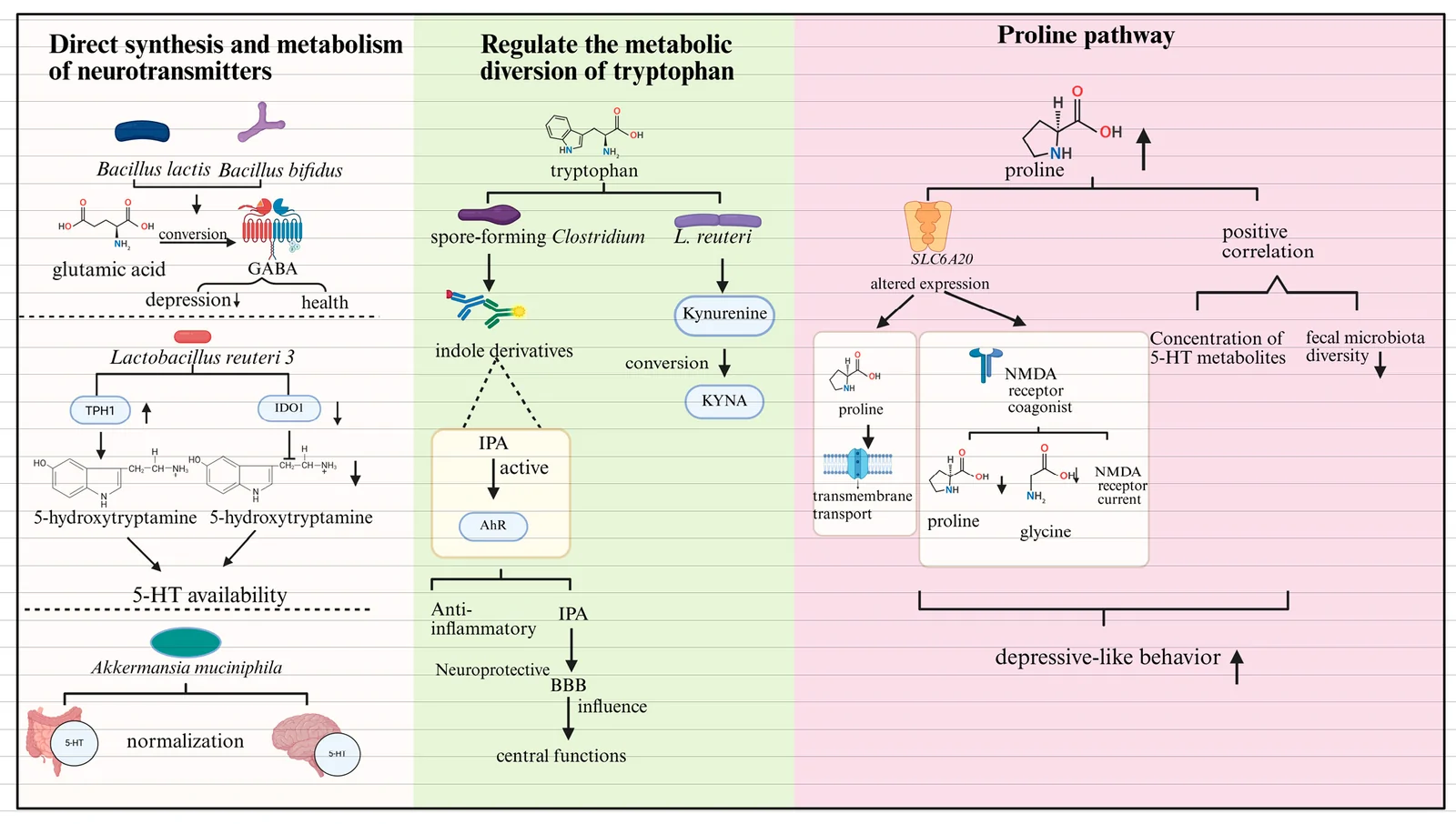
The gut microbiota indirectly modulates central neurotransmitter levels through the generation or metabolism of neuroactive substances. The gut microbiota influences central neurotransmitter levels through three main pathways: (1) Direct synthesis and metabolism of neurotransmitters: *Lactobacillus* and *Bifidobacterium* convert glutamate into GABA; *Lactobacillus reuteri* 3 enhances 5-HT availability via dual mechanisms by upregulating TPH1 to promote 5-HT biosynthesis and inhibiting IDO1 to reduce 5-HT degradation through the kynurenine pathway; and *Akkermansia muciniphila* simultaneously normalizes 5-HT metabolism in both the brain and intestine. (2) Regulation of tryptophan metabolic diversion: spore-forming *Clostridium* species metabolize tryptophan into indole derivatives such as indole-3-propionic acid (IPA), which activates the aryl hydrocarbon receptor (AhR) to exert anti-inflammatory and neuroprotective effects and can cross the blood-brain barrier to directly influence central functions; *L. reuteri* modulates 5-HT availability through the kynurenine-KYNA pathway. (3) The proline pathway: elevated circulating proline levels correlate with depression severity; fecal microbiota transplantation (FMT) alters SLC6A20 expression, affecting proline transmembrane transport and the availability of glycine, an NMDA receptor coagonist, thereby modulating NMDA receptor currents. Clinical studies have demonstrated a positive correlation between fecal microbiota diversity and urinary 5-HT metabolite concentrations. Overall, gut dysbiosis may contribute to emotional dysregulation by disrupting the gut–brain chemical signaling network, although specific causal pathways in humans remain to be validated. GABA: gamma-aminobutyric acid; 5-HT: 5-hydroxytryptamine; BBB: blood-brain barrier.

Gut microbes are capable of indirectly modulating central neurotransmitter concentrations via the biosynthesis and metabolic transformation of neuroactive compounds [9]. On the one hand, experimental evidence from animal models has indicated that select *Lactobacillus* and *Bifidobacterium* strains have the capacity to convert glutamate to γ-aminobutyric acid (GABA) enzymatically; however, diminished cerebral GABA levels have been documented in a subset of patients with depression relative to their healthy counterparts [10,11]. Emerging evidence has further demonstrated that *Lactobacillus reuteri 3* not only upregulates TPH1 expression in the colon, facilitating serotonin (5-HT) biosynthesis, but also attenuates the kynurenine pathway through downregulation of IDO1, consequently diminishing tryptophan diversion toward the kynurenine arm and concomitantly increasing 5-HT production, thereby achieving dual potentiation of 5-HT bioavailability [12,13]. Furthermore, *Akkermansia muciniphila* has been shown to normalize 5-HT metabolism concurrently in both the brain and the gut, suggesting that its antidepressant-like effects may be mediated, at least in part, through bidirectional signaling along the gut–brain axis [14]. Collectively, at least 13 depression-associated taxa have been identified as participants in the synthesis of GABA, glutamate, butyrate, and homovanillic acid, highlighting that the gut microbiota’s influence on neurochemical homeostasis is a systemic, multi-strain synergistic process rather than a consequence of individual microbial actions [15]. On the other hand, the gut microbiota can regulate the metabolic shunting of tryptophan: certain bacteria, such as *Clostridium sporogenes*, convert tryptophan into indole derivatives [16]. Among these, indole-3-propionic acid (IPA) can exert anti-inflammatory and neuroprotective effects through the activation of the aryl hydrocarbon receptor (AhR) and other receptors and is capable of crossing the blood–brain barrier to directly influence central nervous system function [17,18]; alternatively, IPA may affect serotonin (5-HT) availability via the kynurenine pathway [19,20,21]. In addition, elevated circulating and dietary proline levels have been associated with depression severity and have been shown to induce depressive-like behaviors in mice, accompanied by altered expression of proline transport-related genes (Slc6a20) in the brain, suggesting that the involvement of amino acid metabolic pathways in depression may be more extensive than previously recognized [22]. Recent studies have revealed that the proline transporter SLC6A20 not only mediates proline transmembrane transport but also co-transports glycine, a co-agonist of the NMDA receptor; aberrant expression of this transporter leads to reduced cerebral proline and glycine levels, as well as attenuated NMDA receptor currents, implicating the proline transporter in the pathophysiology of depression through the modulation of excitatory neurotransmission [23]. In patients with depression, fecal microbial diversity has been positively correlated with urinary concentrations of 5-HT metabolites in observational studies [24]. In animal experiments, germ-free mice exhibit reduced hippocampal 5-HT and dopamine levels along with prolonged immobility time in the forced swim test [25,26], and recipient mice receiving fecal microbiota transplants from depressed patients display similar alterations [27]. Collectively, these findings suggest that gut dysbiosis may participate in the dysregulation of emotional states by interfering with the gut–brain chemical signaling network; however, the specific causal pathways in humans remain to be validated. Most gut-derived neurotransmitters themselves are unlikely to cross the blood–brain barrier in sufficient amounts due to the restrictive nature of the blood–brain barrier (BBB) and the presence of efflux transporters [28]. Therefore, their influence on the brain is more plausibly mediated through precursors, microbial metabolites, or by acting on enterochromaffin cells to release host-derived signaling molecules [29,30,31]. Furthermore, the kynurenine pathway metabolites can cross the BBB via the large neutral amino acid transporter (LAT1) [32], and their balance is influenced by gut microbiota composition [33]. Thus, the functional relevance of microbial neuroactive compounds in the brain remains an area requiring direct mechanistic validation (Figure 1).

### 2.2. Elicitation of Neuroinflammatory Responses

**Figure 2:**
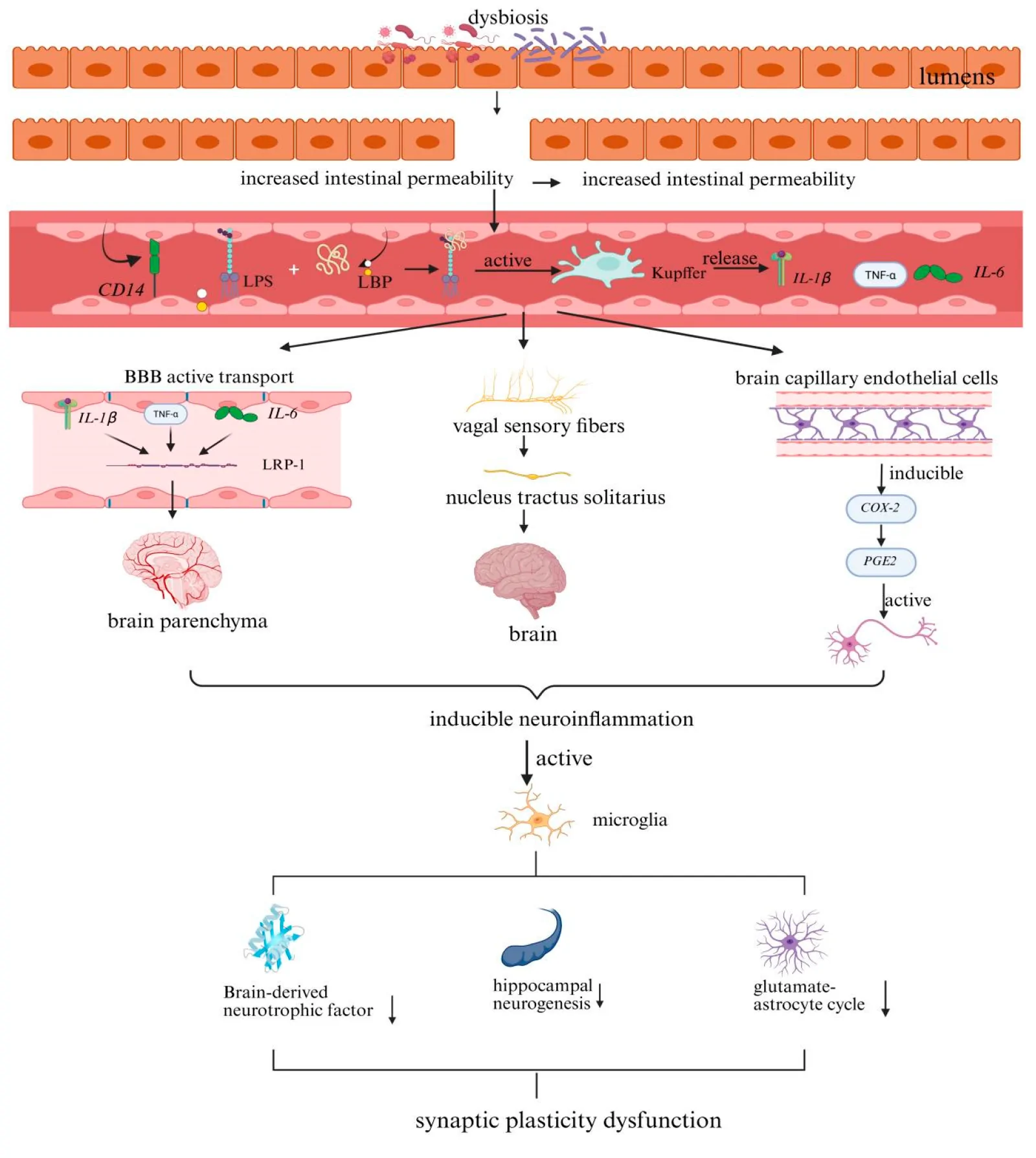
Gut microbiota dysbiosis increases intestinal permeability, facilitating the translocation of lipopolysaccharide (LPS) into the circulation. Upon binding to LPS-binding protein (LBP), LPS activates Kupffer cells via CD14, triggering the release of proinflammatory cytokines, including IL-1β, TNF-α, and IL-6. These inflammatory mediators affect the central nervous system through three parallel pathways: active transport across the blood-brain barrier mediated by LRP-1 into the brain parenchyma; transmission via vagal sensory fibers to the nucleus tractus solitarius; and induction of COX-2 expression in brain capillary endothelial cells, leading to PGE2 production and subsequent neuronal activation. Collectively, these pathways induce neuroinflammation, which activates microglia and ultimately results in reduced brain-derived neurotrophic factor (BDNF), impaired hippocampal neurogenesis, and disruption of the glutamate-astrocyte cycle, culminating in synaptic plasticity dysfunction. CD-14: cluster of differentiation 14, LPS: lipopolysaccharide, LBP: LPS-binding protein, IL-1β: interleukin-1 beta, IL-6: interleukin-6, BBB: blood-brain barrier.

A subset of patients with depression—particularly those presenting with elevated inflammatory markers—exhibits low-grade systemic inflammation that has been linked to gut dysbiosis [34]. Microbial imbalance can increase intestinal epithelial permeability, facilitating the translocation of bacterial products such as lipopolysaccharide (LPS) into the circulation, which in turn activates peripheral immune cells, including hepatic Kupffer cells, to release pro-inflammatory cytokines such as interleukin-1β (IL-1β), tumor necrosis factor-α (TNF-α), and IL-6 [35,36]. These peripheral pro-inflammatory mediators can access the central nervous system via active transport across the blood–brain barrier, vagal afferent pathways, or by inducing cerebral endothelial cells to synthesize prostaglandin E2, thereby activating microglia and eliciting neuroinflammatory responses [37,38]. In preclinical models, established neuroinflammation has been shown to suppress brain-derived neurotrophic factor (BDNF) expression, reduce hippocampal neurogenesis, and interfere with the glutamate–astrocyte cycle, ultimately impairing synaptic plasticity [39,40]. In human observational studies, serum levels of LPS-binding protein and soluble CD14 have been reported to be elevated in depressed patients, suggesting increased endotoxin exposure [41]. Moreover, positron emission tomography (PET) imaging has revealed increased binding of microglial activation markers in the anterior cingulate cortex of a subset of patients [42]. However, whether neuroinflammation represents a cause or a consequence of depression remains a matter of debate, and it may constitute a pathological feature specific to certain disease subtypes rather than a universal hallmark of the disorder (Figure 2).

### 2.3. Impairment of Intestinal Epithelial Barrier Integrity

The intestinal barrier serves as the first physical line of defense in gut–brain homeostasis, comprising intestinal epithelial cells and intercellular tight junction proteins (occludin, claudin, and ZO-1), whose integrity directly determines whether bacterial products can gain access to the systemic circulation [43]. Gut dysbiosis can compromise this barrier through two complementary pathways. First, structural disruption—an increase in pathogenic strains or a reduction in commensal bacteria—can directly impair tight junction integrity, leading to increased intestinal epithelial permeability and resulting in increased intestinal permeability [44]. Second, metabolic imbalance—a decrease in short-chain fatty acid (SCFA)-producing bacteria—results in an insufficient supply of substrates such as butyrate, which are essential for maintaining epithelial barrier function [45]. Concurrently, lipopolysaccharide (LPS) released by Gram-negative bacteria can induce the degradation of tight junction proteins, creating a vicious cycle in which dysbiosis elevates LPS levels, which in turn disrupts epithelial junctional complexes and increases intestinal permeability, ultimately compromising barrier function. Once the barrier is breached, substantial amounts of LPS enter the circulation and trigger neuroinflammation [44]. Human studies have provided direct evidence for this cascade. In cross-sectional human studies, serum levels of zonulin—the only known endogenous factor capable of reversibly modulating intestinal epithelial tight junction permeability—have been reported to be elevated in patients with depression and associated with intestinal barrier dysfunction [46,47]. Transdiagnostic studies have further revealed increased serum claudin-5 and zonulin in patients with bipolar disorder [48], suggesting that intestinal barrier impairment may represent a shared feature across psychiatric disorders. In animal experiments, chronic stress or transplantation of fecal microbiota from depressed patients has been shown to reduce tight junction protein expression and increase intestinal permeability, whereas probiotic supplementation or fecal microbiota transplantation ameliorates these alterations [49,50]. Collectively, these findings indicate that dysbiosis, through disruption of both the physical and chemical defenses of the intestinal barrier, creates permissive conditions for LPS and pro-inflammatory cytokines to enter the systemic circulation, thereby constituting a critical initiating event in gut–brain axis dysregulation and serving as a bridge linking peripheral inflammation to central pathology.

### 2.4. Modulation of Neuroendocrine Signaling via the HPA Axis

The gut microbiota serves as a critical modulator of hypothalamic–pituitary–adrenal (HPA) axis function, and hyperactivation of the HPA axis represents one of the core neuroendocrine features of depressive disorders [51]. Animal experiments have provided causal evidence for this relationship: compared with conventionally housed mice, germ-free mice present significantly elevated basal levels of adrenocorticotropic hormone (ACTH) and corticosterone; however, these levels normalize following colonization with a normal microbiota, suggesting that microbiota depletion leads to blunted HPA axis negative feedback [51]. Further studies have identified specific strains, such as *Lactobacillus reuteri*, as key species involved in restoring HPA axis circadian rhythmicity [52]. Microbiota depletion disrupts the circadian secretion pattern of glucocorticoids, whereas supplementation with this strain or fecal microbiota transplantation can reestablish this rhythm. In patients with depression, HPA axis hyperactivation manifests as chronic cortisol elevation, downregulation of glucocorticoid receptor (GR) expression in the hippocampus and pituitary gland, and reduced negative feedback sensitivity [53,54]. The microbiota can modulate HPA axis activity through three principal pathways. First, microbial metabolites such as short-chain fatty acids (SCFAs) regulate the excitability of corticotropin-releasing hormone (CRH) neurons in the hypothalamic paraventricular nucleus (PVN) by activating free fatty acid receptors (FFAR2/FFAR3) or by directly entering the circulation [55,56]. Second, vagal afferent fibers transmit intestinal inflammatory signals to the nucleus tractus solitarius (NTS), which projects to the PVN via relay in the parabrachial nucleus, thereby activating CRH neurons [57]. Third, systemic inflammatory cytokines (IL-1β and IL-6) can act directly on the hypothalamus to promote CRH release. Notably, HPA axis activation and intestinal barrier disruption form a vicious cycle: elevated cortisol levels can increase intestinal epithelial permeability, facilitating bacterial translocation and inflammatory responses, while inflammation in turn further drives HPA axis hyperactivation [55,58]. Collectively, the microbiota participates in the endocrine pathophysiology of depression through a “microbiota–HPA axis–intestinal barrier” circuit, and targeting this circuit may offer novel therapeutic strategies for the management of depressive disorders.

### 2.5. Influence on Vagal Afferent Signaling Pathways

The vagus nerve constitutes the principal direct neural communication route of the “microbiota–gut–brain” axis, with approximately 80% of its fibers being afferent in nature and responsible for transmitting visceral signals from the gastrointestinal tract to the central nervous system [59]. Enteroendocrine cells (EECs) form synapse-like connections with vagal nerve terminals through specialized “neuropods” [60], enabling microbial metabolites—including short-chain fatty acids (SCFAs), neurotransmitters, and peptide hormones—to directly activate vagal afferent fibers, which then relay signals to the brainstem nucleus tractus solitarius (NTS), with subsequent projections to emotion-regulating brain regions such as the amygdala, insula, and prefrontal cortex [61]. Animal experiments have provided direct causal evidence for this pathway: germ-free mice exhibit significantly reduced vagal nerve activity compared with conventional mice, and colonization with a normal microbiota restores vagal activity to normal levels; antibiotic treatment decreases vagal activity in normal mice, yet this effect can be reversed by lavage with intestinal luminal fluid (containing microbial metabolites) from normal mice, whereas luminal fluid from germ-free mice fails to produce such an effect [62]. More importantly, subdiaphragmatic vagotomy (SDV) has been shown to block depressive-like behaviors in multiple depression models, including LPS injection [63], fecal microbiota transplantation from depressed patients [64], Chrna7 gene knockout, and chronic stress paradigms [65,66], in all cases demonstrating that depressive phenotypes cannot be induced in the absence of vagal afferent input, thereby establishing the vagus nerve as an indispensable pathway for the central transmission of microbial signals [67]. In addition, the vagus nerve regulates the cholinergic anti-inflammatory pathway (CAIP): various fibers release acetylcholine, which acts on α7 nicotinic acetylcholine receptors (α7 nAChRs) on immune cells such as macrophages, thereby inhibiting NF-κB activation and the release of pro-inflammatory cytokines [68]. Patients with depression have been reported to exhibit reduced vagal tone, as reflected by decreased heart rate variability in observational studies, which may compromise the anti-inflammatory reflex and exacerbate systemic inflammation [69,70]. Invasive vagus nerve stimulation (VNS) was approved by the US Food and Drug Administration (FDA) in 2005 as an adjunctive long-term treatment for chronic or recurrent treatment-resistant depression in patients with at least four failed antidepressant trials. By contrast, transcutaneous auricular vagus nerve stimulation (taVNS) remains an investigational, non-invasive alternative that has not received regulatory approval for depression; its clinical use in treatment-resistant depression is supported mainly by small, open-label or preliminary trials, and larger sham-controlled studies are still needed to establish efficacy and standardize stimulation parameters. Where antidepressant effects of taVNS have been reported, they are thought to be partially attributable to enhanced vagal tone and reduced peripheral and central inflammation [71,72]. In summary, the vagus nerve serves as the core information superhighway of the “microbiota–gut–brain” axis, functioning both as an anatomical substrate for the ascending transmission of microbial signals and as a functional hub for anti-inflammatory protection and emotional regulation, thereby offering a novel target for neuromodulation-based therapeutic interventions in depression.

## 3. Impact of Factors That Influence Depression on the Gut Microbial Composition

Dietary patterns and psychological stress represent two pivotal determinants in the development and course of depressive disorders, each of which may be further parsed into a spectrum of downstream variables, including sleep and other lifestyle factors. Notably, both dietary intake and stress exposure have been shown to profoundly shape the compositional architecture of the gut microbial community.

### 3.1. Possible Impact of Depressive Disorders on the Gut Microbiota Composition

Patients with major depressive disorder (MDD) exhibit alterations in gut microbial composition compared with healthy controls, particularly with respect to microbial diversity and the abundance of certain anti-inflammatory bacterial taxa, with overall gut microbial richness being reduced [73]. In addition, references therein. In contrast, differences in both the alpha and beta diversity of the gut microbiota have been reported between depressed patients and healthy individuals. At the phylum level, findings regarding the abundance of Firmicutes, Bacteroidetes, and Proteobacteria have been inconsistent across studies: some cohorts report an increased relative abundance of Bacteroidetes or a decreased abundance of Firmicutes, whereas others find no significant difference or report opposite trends. This inconsistency likely reflects differences in study populations, geographic regions, sequencing platforms, and analytical pipelines, as well as the confounding effects of antidepressants and diet. Notably, however, the Bacteroidetes/Firmicutes ratio—a composite index rather than the abundance of either phylum alone—has been more consistently reported to be increased in MDD patients [74], primarily manifesting as enrichment of the genus Bacteroides and depletion of Blautia, Faecalibacterium, and Coprococcus. This distinction is important: the relative robustness of the ratio does not imply that the directional changes of the two phyla are themselves consistent across individual studies, and the ratio should therefore be interpreted with caution rather than as a definitive microbial signature of MDD. In addition, an increased abundance of Eggerthella and a decreased abundance of Sutterella have been consistently observed in MDD patients. However, the gut microbial composition in depressed patients also varies with age and geographic region. For example, in certain Chinese patient cohorts, increased Eggerthella along with decreased Coprococcus and Fusicatenibacter have been reported [3].

**Figure 3:**
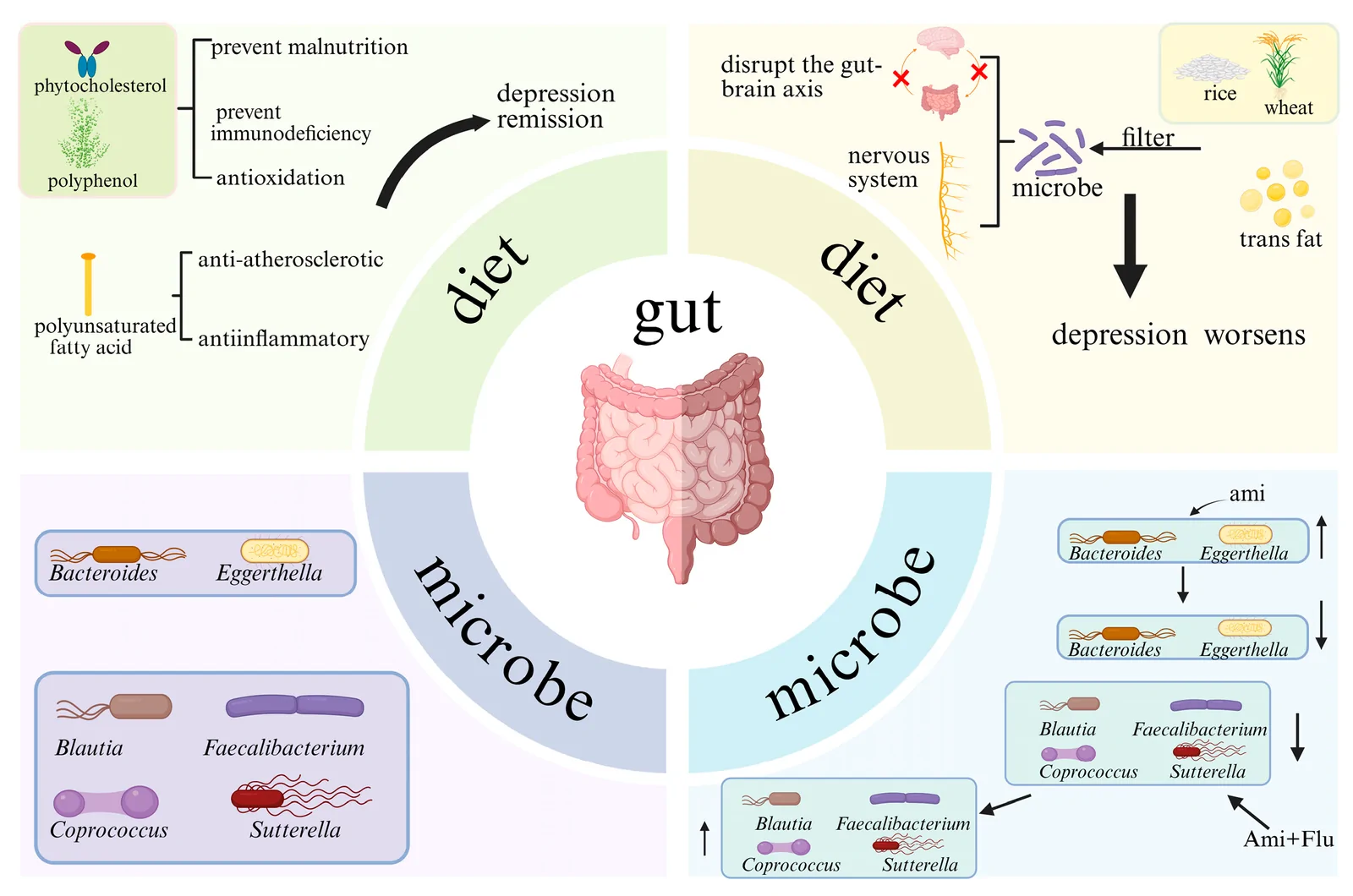
Interplay among diet, the gut microbiota, and depression. This schematic illustrates how dietary components and intestinal microbes influence depression onset and remission through distinct pathways. (**Upper left**) A healthy diet rich in phytosterols, polyphenols, and polyunsaturated fatty acids promotes depression remission via antioxidant, anti-inflammatory, anti-atherosclerotic, anti-malnutrition, and immunomodulatory effects. (**Upper right**) An unhealthy diet characterized by refined carbohydrates (rice and wheat) and trans fats disrupts the gut-brain axis, impairs nervous system function, and alters microbial composition, thereby exacerbating depressive symptoms. (**Lower left**) Depression remission is associated with enrichment of specific gut bacteria, including *Bacteroides*, *Eggerthella*, *Blautia*, *Faecalibacterium*, *Coprococcus*, and *Sutterella*. (**Lower right**) Antibiotic (Ami) and/or fluoxetine (Flu) treatment modulates the gut microbiota structure; changes in the abundance of *Blautia*, *Faecalibacterium*, *Coprococcus*, and *Sutterella* correlate with therapeutic efficacy, suggesting these taxa as potential biomarkers for treatment response in depression. Ami: amitriptyline; Flu: fluoxetine.

Antidepressant medications, such as fluoxetine (Flu) and escitalopram, can reduce gut microbial abundance, particularly affecting genera such as Ruminococcus, *Adlercreutzia*, and unclassified *Alphaproteobacteria*, which may contribute to constipation; prolonged use of these agents may induce alterations in synaptic and mitochondrial gene expression as well as changes in monoamine neurotransmitter levels, thereby attenuating antidepressant efficacy and leading to drug resistance [8]. Treatment with fluoxetine and the tricyclic antidepressant amitriptyline (Ami) has been shown to reduce the *Firmicutes*/*Bacteroidetes* ratio and improve neurological outcomes [75]. At the genus level, rats receiving amitriptyline (Ami) and fluoxetine (Flu) presented significantly greater relative abundances of *Bacteroides*, *Parabacteroides*, and *Butyricimonas* in fecal samples than did those exposed to unpredictable chronic stress paradigms, an observation that implicates these microbial taxa and their metabolic products in the maintenance of brain health [76] (Figure 3, Table 1).

### 3.2. Impact of Dietary Intake on the Gut Microbial Composition in Patients with Depression

Researchers have compared the dietary patterns of patients with depression—which tend to resemble a Western-style diet—with those of healthy individuals, who typically adhere more closely to a Mediterranean diet [80]. The diet of depressed patients is characterized by high intakes of refined carbohydrates and trans fats, which can rapidly select for microbial communities adapted to such nutritional substrates, thereby altering the gut microbial composition and disrupting the intestinal environment [81]. These diet-selected microbial shifts may, in turn, reinforce sustained consumption of the same food types and contribute to disturbances across multiple physiological systems, including the gut–brain axis, gastrointestinal function, dietary metabolism, circulatory homeostasis, immune competence, and endocrine regulation, ultimately affecting the nervous system and either predisposing individuals to or exacerbating depressive disorders [77]. In contrast, the dietary pattern of healthy individuals—typically aligned with the Mediterranean diet—emphasizes natural, minimally processed components, providing polyunsaturated fatty acids (found in olive oil and nuts) with anti-atherogenic and anti-inflammatory properties, as well as bioactive compounds endowed with antioxidant activity, such as flavonoids, phytosterols, terpenes, and polyphenols [82]. This dietary framework is also replete with micronutrients that safeguard against nutritional insufficiency and immune compromise. Furthermore, the ketogenic diet, characterized by carbohydrate restriction with commensurate increases in fat and protein intake, has demonstrated anxiolytic- and antidepressant-like efficacy in preclinical models, suggesting therapeutic potential for neuropsychiatric conditions [83]. In summary, dietary patterns exert a pronounced influence on the gut microbial constitution, the repercussions of which transcend depression itself: perturbation of the intestinal ecosystem can engender systemic derangements predisposing individuals to diverse somatic pathologies and neuroinflammation, thereby offering a mechanistic explanation for the frequent co-occurrence of comorbid conditions in patients with depressive disorders. Because severe depressive symptoms may affect appetite, meal regularity, and physical activity, clinical interpretation of microbiome findings should also consider diet, physical activity, sleep, and medication exposure as potential confounders [84]. For example, reduced physical activity is associated with a lower abundance of butyrate-producing genera (*Faecalibacterium*, *Roseburia*), while higher exercise levels correlate with increased *Lactobacillus* and *Bifidobacterium* [85,86,]. Moreover, MDD patients often exhibit irregular meal timing and higher consumption of ultra-processed foods, which may synergistically exacerbate microbial dysbiosis and inflammation [87,88]. A more comprehensive assessment of patients’ dietary habits, including caloric intake, macronutrient composition, and meal frequency, is warranted in future studies to clarify the diet–microbiome–depression triad [89].

### 3.3. Effects of Stress Dysregulation in Depressed Patients on the Gut Microbiome

Both acute and chronic stress exert profound effects on the brain–gut–microbiome axis, with stress responses capable of inducing rapid alterations in the gut microbial composition and eliciting intestinal inflammation, in which the enteric nervous system (ENS) plays a pivotal role in amplifying inflammatory cascades [90]. Initially, it was believed that stress-induced adrenal release of glucocorticoids directly targeted intestinal immune cells to provoke inflammation; however, subsequent investigations revealed that glucocorticoids act on enteric neurons, which in turn connect with microglia in the brain, thereby activating intestinal immunity and leading to gut inflammation. Concurrently, glucocorticoids can attenuate the molecular signals that govern muscle contraction, thereby slowing intestinal motility and peristalsis and exacerbating inflammatory discomfort [91]. In animal models of stress dysregulation, altered abundances of *Firmicutes* and *Tenericutes* have been consistently observed in chronic mild stress (UCMS) models, with a marked reduction in *Lactobacillus* in the *Firmicutes* phylum. In chronic social defeat stress (CSDS) models, increases in *Bacteroidetes* and *Spirochaetes* have been reported, primarily reflected in compositional changes within the *Ruminococcaceae* family. In social overcrowding (SOC) models, increased relative abundances of *Akkermansia muciniphila* and *Anaerostipes*, in addition to decreased abundances of *Erysipelotrichaceae*, *Lactobacillus*, and *Acidobacteroides*, have been documented [92]. The hypothalamic–pituitary–adrenal (HPA) axis coordinates the release of hormones such as cortisol in response to stress [93]. Stress signals—primarily in the form of neural impulses—are transmitted through the sympathetic nervous system and the HPA axis, activating both the hypothalamic–pituitary–adrenal system and the sympathetic–adrenal–medullary (SAM) axis and resulting in the release of epinephrine and norepinephrine, as well as glucocorticoid secretion, which suppresses immune responses [94]. Stress mediators such as norepinephrine can activate microbial genes, increasing bacterial contact with the intestinal immune system and promoting intestinal inflammation [95,96]. Prolonged stress dysregulation leads to disruption of the kynurenine pathway; indeed, dysregulation of tryptophan metabolites—which are directly linked to brain function and neurological disorders, including depression and Alzheimer’s disease—has been observed across multiple brain disorders [97,98]. In the gastrointestinal tract, kynurenine is synthesized from tryptophan via the enzyme indoleamine-2,3-dioxygenase (IDO), a process that is heavily influenced by Firmicutes in regulating serotonin synthesis in enterochromaffin cells; *Lactobacillus* specifically determines the extent to which tryptophan is shunted toward the kynurenine pathway [98]. Stress-induced reduction in *Lactobacillus* and concomitant increase in IDO activity result in elevated kynurenine production. As kynurenine competes with tryptophan for transport across the blood–brain barrier, increased intestinal kynurenine production reduces the availability of tryptophan for cerebral serotonin synthesis. Unlike serotonin, however, kynurenine can freely cross from the blood into the brain [99]. An elevated kynurenine-to-serotonin ratio in the brain has been associated with neuroinflammation and neurodegeneration and has also been implicated in the pathogenesis of certain forms of depression [100]. Furthermore, fecal microbiota transplantation experiments have demonstrated that the gut microbiota is essential for mounting immune and serotonergic responses to intestinal stress during both acute and chronic stress paradigms [101].

In summary, patients with depression frequently present with aberrant dietary habits and sustained stress dysregulation that remain unresolved, leading to gut dysbiosis, metabolic disturbances, and disruption of the intestinal–brain internal milieu. These alterations predispose individuals to the development of comorbid medical conditions; compromise immune, neural, and circulatory homeostasis; and interfere with gut–brain axis signaling, ultimately impairing brain health and establishing a vicious cycle that perpetuates the onset and progression of depressive disorders.

## 4. Therapeutic and Preventive Potential of the Gut Microbiome in Depression

As a common psychiatric condition, depression has garnered increasing attention because of its close relationship with the intestinal system, which exerts modulatory effects on depressive pathophysiology via diverse routes encompassing neural, immunological, and humoral mechanisms. Concurrently, an expanding body of research has underscored the functional involvement of the gut microbiota in the therapeutic and prophylactic management of depression.

### 4.1. Antidepressant Medications and Gut Microbiome Interactions in Depression

Pharmacological intervention currently constitutes the primary therapeutic approach for treating depressive disorders. Medications can alter bacterial metabolism through their actions on the gut microbiota, and their activity and efficacy are in turn significantly influenced by the composition and functional capacity of the gut microbial ecosystem [102]. Clinically used antidepressants possess antimicrobial properties, and agents such as selective serotonin reuptake inhibitors (SSRIs) and tricyclic antidepressants have been shown to modify microbial diversity and composition [8]. SSRI treatment increases the abundance of Eubacterium ramulus in the gut, whereas tricyclic antidepressants are associated with an increased abundance of *Clostridium leptum* [103]. Moreover, duloxetine substantially increases (by more than 100-fold) the abundance of Eubacterium rectale [104]. Given that these bacterial taxa are known to produce anti-inflammatory butyrate during their metabolic activities, their increased abundance may exert an adjuvant effect on antidepressant pharmacotherapy, thereby enhancing therapeutic efficacy [3]. Furthermore, a prospective cohort study indicated that the gut microbiota may serve as a predictor of antidepressant treatment outcomes in elderly depressed patients [2]: relative to *Lachnoclostridium* (as the reference standard), enrichment of *Faecalibacterium*, *Roseburia*, and *Agathobacter* at baseline was associated with remission of depressive symptoms [3]. In addition, psychobiotics have been demonstrated to improve mood, reduce anxiety, and enhance cognitive function in both healthy individuals and patients [105]. Medicinal plants have been proposed as optimal sources of psychobiotic compounds; these plants contain complex mixtures of bioactive constituents, many of which exhibit low oral bioavailability. Some of these compounds are poorly absorbed in the upper gastrointestinal tract because of their relatively high molecular weight and polarity, which may facilitate their uptake by other cells and promote the formation of a healthy enterotype, thereby reducing the *Bacteroidetes*/*Firmicutes* ratio and ameliorating the progression of depression [3,100]. However, it should be noted that various pathogenic bacteria—including *Streptococcus salivarius*, *Bifidobacterium uniformis*, *Bifidobacterium* species, and *Escherichia coli* IAI1—have been shown to increase the bioaccumulation of duloxetine, which may reduce its bioavailability and consequently diminish its therapeutic efficacy [3].

### 4.2. Potential Therapeutic Effects of Probiotics in Depression Management

Probiotics have been found to modulate the gut microbial balance, thereby conferring benefits to host health. As the most extensively studied microbiota-targeted therapy for depression, probiotics typically involve combinations of strains from the genera *Lactobacillus* and *Bifidobacterium*. In in vitro and animal studies, these microorganisms have been shown to suppress inflammation and regulate immune responses by preventing induction of the pro-inflammatory cytokine IL-8 in human colonic epithelial cells, while also reducing intestinal permeability and inhibiting endotoxemia. Administration of the probiotic strain *Bifidobacterium infantis* to rats has been reported to reverse experimentally induced stress and depressive-like behaviors [106]. A comprehensive study identified 178 probiotic species and subspecies with potential antidepressant effects; notably, the majority of probiotic formulations currently employed in depression management are based on select species of the genera *Lactobacillus* and *Bifidobacterium* [3]. Similarly, the probiotics fructo-oligosaccharides (FOSs) and galacto-oligosaccharides (GOSs), along with certain medicinal plants, represent the best-documented prebiotic interventions in the context of depression, although their clinical benefit has not been consistently reproduced [3]. Collectively, current evidence is insufficient to recommend probiotics as a standalone or preventive treatment for depression, and they should be regarded as adjunctive or investigational approaches pending further validation [106] (Table 2).

### 4.3. Potential Preventive Relevance of Lactobacillus Species in Anxiety and Depressive Disorders

**Figure 4:**
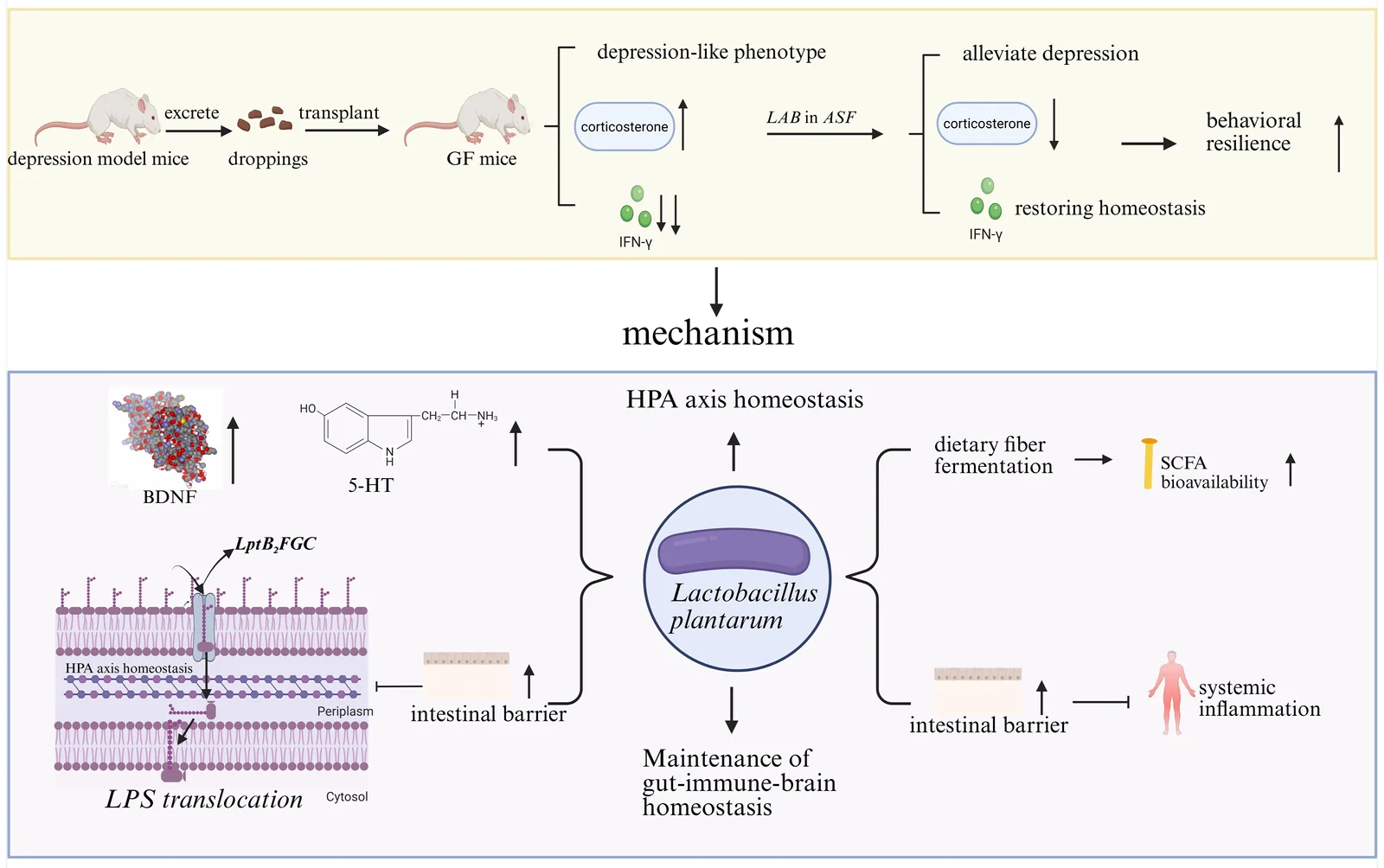
Schematic overview of the gut-immune-brain axis mechanism underlying the potential antidepressant effects of *Lactobacillus plantarum*. Fecal transplantation from depression model mice into germ-free (GF) mice induces a depression-like phenotype characterized by elevated corticosterone and reduced IFN-γ levels. Supplementation with *Lactobacillus* (LAB) in the antidepressant formula (ASF) reduces depression-like behavior by normalizing corticosterone and restoring IFN-γ homeostasis, ultimately enhancing behavioral resilience. The underlying mechanism involves *Lactobacillus plantarum* maintaining gut–immune–brain homeostasis through multiple pathways: strengthening intestinal barrier integrity via the LptB_2_FGC complex to inhibit LPS translocation, thereby restoring HPA axis homeostasis and promoting brain-derived neurotrophic factor (BDNF) and serotonin (5-HT) expression; enhancing dietary fiber fermentation to increase short-chain fatty acid (SCFA) bioavailability; and reinforcing intestinal barrier function to attenuate systemic inflammation. These coordinated mechanisms collectively sustain gut–immune–brain axis homeostasis and exert antidepressant effects. IFN-γ: interferon-gamma; BDNF: brain-derived neurotrophic factor; 5-HT: 5-hydroxytryptamine; HPA: hypothalamic-pituitary-adrenal; SCFA: short-chain fatty acid.

*Lactobacillus* can attenuate susceptibility to depression and anxiety by upregulating the immune mediator interferon-γ (IFN-γ), thereby reshaping the immune–neuroendocrine response to stress [112]. Experimental evidence has demonstrated that transplantation of the gut microbiota from depression-model mice into germ-free (GF) recipients results in a depressive phenotype, accompanied by elevated corticosterone levels and a marked reduction in IFN-γ. In GF mice lacking *Lactobacillus*, basal IFN-γ levels are even lower, and the stress-induced decline in IFN-γ is most pronounced. Reconstitution with *Lactobacillus* strains from the altered Schaedler Flora restores IFN-γ homeostasis and enhances behavioral resilience [112]. Human and animal data further support this pathway. In observational analyses, the DSSS-PS pain score was strongly positively correlated with Slackia (r ≈ 0.93, *p* < 0.001) and positively correlated with the genera *Lactobacillus* and Bifidobacterium and negatively correlated with Sutterella, suggesting that increased *Lactobacillus* abundance may be indirectly associated with reduced depression risk via inhibition of pain sensitization. In an 8-week open-label trial, *Lactobacillus plantarum* PS128 improved mood scores and physiological stress indicators in patients with major depressive disorder [113]; however, the absence of a placebo control limits interpretation. Mechanistically, *Lactobacillus* can promote the fermentation of dietary fiber, thereby increasing the bioavailability of neuroactive metabolites such as short-chain fatty acids; strengthen the intestinal barrier, thereby reducing LPS translocation and systemic inflammation; and modulate 5-HT, BDNF, and HPA axis activity, providing multi-target support for the maintenance of gut–immune–brain homeostasis [112]. In summary, *Lactobacillus* not only serves as a guardian of intestinal health but also represents a potential microbiome-based intervention target for the prevention of depression. However, its effects appear to be strain-specific, and the current evidence is largely derived from animal studies or open-label human trials without placebo control. Controlled randomized trials with larger sample sizes, standardized strains and dosages, and long-term follow-up are necessary to confirm any prophylactic or therapeutic role, and negative or equivocal placebo-controlled results reported for other probiotic strains should temper expectations regarding *Lactobacillus*-based interventions (Figure 4).

## 5. Current Issues, Methodological Recommendations, and Future Directions

In terms of therapeutics, many researchers are actively expanding the frontiers of investigation by developing novel antidepressant agents, exploring targeted therapies, and examining the potential of single compounds to intervene across multiple therapeutic pathways. Future treatment strategies should place greater emphasis on the complex interactions among bacteria, between bacteria and metabolites, and between microorganisms and the host. At present, the clinical diagnostic criteria for depression lack well-defined boundaries, therapeutic options remain limited with only modest efficacy, and a substantial proportion of patients are refractory to conventional antidepressants, rendering complete remission difficult to achieve [114]. The mechanisms by which probiotics—such as *Lactobacillus*—exert their effects on mood regulation and depression intervention remain incompletely elucidated, and related research remains insufficient. Moreover, because probiotic effects are strain-specific and placebo-controlled trials have yielded inconsistent results, findings obtained with one strain or formulation cannot be extrapolated to others. Nevertheless, probiotics are increasingly recognized as potential therapeutic modalities. Future efforts should also pay greater attention to the bidirectional regulatory mechanisms between the gut and the brain and rigorously investigate the role of probiotics and prebiotics in promoting overall health and psychological well-being [115].

Currently, our understanding of how the microbiota modulates brain function through the gut–brain axis remains limited [116]. There is an urgent need for systematic investigations into the associations between the microbiome and disease symptomatology, as well as the bidirectional mechanistic pathways involving immune, neural, and metabolic factors [115]. Research on probiotics and prebiotics should be intensified, with isotope labeling and tracing techniques employed to elucidate the mechanistic roles of key molecular and metabolic mediators linking the microbiota to the brain [117,118]. In addition, the antidepressant potential of immunomodulatory agents such as non-steroidal anti-inflammatory drugs (NSAIDs) warrants systematic examination, along with in-depth exploration of the immunomodulatory role of acetate and the putative antidepressant mechanisms of propionate and butyrate [119]. Multiple experimental studies have demonstrated the efficacy of combined probiotic and prebiotic formulations (synbiotics) in depression intervention, and future investigations should further explore their application potential in the field of psychobiotics [120]. The ultimate goal is to clarify the mechanistic framework of the gut–brain axis, systematically delineate the causal relationships between microbial communities and depressive symptomatology, and thereby provide a theoretical foundation and practical pathways for precision intervention in psychiatric disorders [121].

## 6. Conclusions

This review summarizes the multiple pathogenic mechanisms by which the gut microbiota participates in depressive disorders through the microbiota–gut–brain axis, encompassing aberrant neurotransmitter metabolism, neuroinflammation, intestinal barrier disruption, hypothalamic–pituitary-adrenal (HPA) axis dysfunction, and vagal nerve signaling. Environmental factors such as dietary patterns and chronic stress have been shown to profoundly reshape the gut microbial composition, whereas antidepressant medications engage in bidirectional interactions with the gut microbiota [122,123]. Probiotics—particularly certain strains of *Lactobacillus* and *Bifidobacterium*—have shown adjunctive therapeutic and potential preventive effects through multiple pathways, including the reinforcement of intestinal barrier integrity, the suppression of inflammation, and the modulation of immune responses; however, these effects are strain-specific, and clinical findings remain heterogeneous, with inconsistencies [124,125]. The mechanisms elucidated in this review are expected to facilitate the clinical translation of microbiota-targeted intervention strategies and provide novel therapeutic avenues for the management of depressive disorders.

## Figures and Tables

**Table 1:** Effects of different factors on the gut microbiota.

Influencing Factor	Study Type	Microbial Strains with Increased Abundance	Microbial Strains with Decreased Abundance	Potential Impact	Reference
Depression itself	Cross-sectional	*Bacteroides*, *Eggerthella*, *Collinsella*, *Lachnoclostridium*	*Faecalibacterium*, *Coprococcus*, *Blautia*, *Sutterella*, *Dialister*	Reduced anti-inflammatory butyrate producers; increased pro-inflammatory taxa	[74]
Antidepressants	Animal model	*Bacteroidetes*, *Parabacteroides*, *Alistipes* (amitriptyline/fluoxetine); *Eubacterium ramulus* (SSRIs); *Eubacterium rectale* (duloxetine)	*Ruminococcus*, *Adlercreutzia*, *Alphaproteobacteria*	Drug–microbiome bidirectional interactions; some taxa may enhance or reduce antidepressant efficacy	[8]
Western diet	Cross-sectional	Microbes favoring refined carbs and trans fats	*Lactobacillus*, *Bifidobacterium*; butyrate producers	Promotes dysbiosis, intestinal permeability, and low-grade inflammation	[77]
Mediterranean diet	Randomized controlled trial	*Bifidobacterium*, *Lactobacillus*, butyrate producers	Pro-inflammatory *Proteobacteria*	Improves depressive symptoms	[78]
Chronic stress	Animal models	*Bacteroidetes*, *Ruminococcaceae*, *Akkermansia*	*Lactobacillus*, *Acidobacteria*, *Erysipelatoclostridiaceae*	HPA axis dysregulation	[79]

**Table 2:** Effects of different probiotic strains on increases or decreases in metabolites or inflammatory factors.

Probiotic Genus/Species	Substances That Increase	Substances That Decrease	References
*Lactobacillus*	GABA, short-chain fatty acids (e.g., butyrate), interferon-γ (IFN-γ)	Interleukin-6 (IL-6), tumor necrosis factor-α (TNF-α)	[107]
*Bifidobacterium*	Butyrate, γ-aminobutyric acid (GABA), interleukin-10 (IL-10)	Lipopolysaccharide (LPS), pro-inflammatory cytokines (e.g., IL-6, TNF-α)	[107]
*Faecalibacterium*	Butyrate, interleukin-10 (IL-10)	Interleukin-6 (IL-6), tumor necrosis factor-α (TNF-α)	[108,109]
*Clostridium* (partial strains)	Propionate, butyrate	p-Cresol, glutathione (GSH) [decreased in some strains]	[108]
*Roseburia*	Butyrate, interleukin-10 (IL-10)	Lipopolysaccharide (LPS)	[110]
*Eubacterium rectale*	Butyrate, anti-inflammatory factors	—	[108]
*Akkermansia*	Mucus layer thickness, short-chain fatty acids	Lipopolysaccharide (LPS)	[111]

## Data Availability

No new data were created or analyzed in this study. Data sharing is not applicable to this article.

## Abbreviations

The following abbreviations are used in this manuscript:

RCT	Randomized controlled trial
FOS	Fructo-oligosaccharides
GOS	Galacto-oligosaccharides
IFN-γ	Interferon-γ
GF	Germ-free
LPS	Lipopolysaccharide
BDNF	Brain-derived neurotrophic factor
HPA	Hypothalamic–pituitary–adrenal
5-HT	5-Hydroxytryptamine/Serotonin
GABA	γ-Aminobutyric acid
TPH1	Tryptophan hydroxylase 1
IDO1	Indoleamine 2,3-dioxygenase 1
IPA	Indole-3-propionic acid
AhR	Aryl hydrocarbon receptor
SLC6A20	Solute carrier family 6 member 20
NMDA	N-methyl-D-aspartate
BBB	Blood–brain barrier
LAT1	L-type/large neutral amino acid transporter 1
IL-1β	Interleukin-1β
TNF-α	Tumor necrosis factor-α
IL-6	Interleukin-6
PET	Positron emission tomography
ZO-1	Zonula occludens-1
SCFA	Short-chain fatty acid
ACTH	Adrenocorticotropic hormone
GR	Glucocorticoid receptor
CRH	Corticotropin-releasing hormone
PVN	Hypothalamic paraventricular nucleus
FFAR2/FFAR3	Free fatty acid receptor 2/3
NTS	Nucleus tractus solitarius
EEC	Enteroendocrine cell
SDV	Subdiaphragmatic vagotomy
CAIP	Cholinergic anti-inflammatory pathway
α7 nAChR	α7 nicotinic acetylcholine receptor
NF-κB	Nuclear factor kappa-B
taVNS	Transcutaneous auricular vagus nerve stimulation
UCMS	Unpredictable chronic mild stress
CSDS	Chronic social defeat stress
SOC	Social overcrowding
ENS	Enteric nervous system
SAM	Sympathetic–adrenal–medullary
SSRI	Selective serotonin reuptake inhibitor
Flu	Fluoxetine
Ami	Amitriptyline
NSAID	Non-steroidal anti-inflammatory drug
FMT	Fecal microbiota transplantation
ASF	Altered Schaedler Flora
LAB	Lactic acid bacteria/Lactobacillus
KYNA	Kynurenic acid
Kyn	Kynurenine
LBP	LPS-binding protein
CD14	Cluster of differentiation 14
COX-2	Cyclooxygenase-2
PGE2	Prostaglandin E2
LRP-1	LDL receptor-related protein 1
IL-10	Interleukin-10
GSH	Glutathione
MDD	Major depressive disorder
MGBA	Microbiota–gut–brain axis
